# Striking a balance in sports: the interrelation between children's sports experience, body size, and posture

**DOI:** 10.3934/Neuroscience.2022016

**Published:** 2022-06-24

**Authors:** Piali Bhati, Theodore C. K. Cheung, Gobika Sithamparanathan, Mark A. Schmuckler

**Affiliations:** Department of Psychology, University of Toronto Scarborough, 1265 Military Trail, Scarborough, ON, Canada, M1C 1A4

**Keywords:** postural control, multisensory influences, sports participation, dynamic systems, developmental processes

## Abstract

This study investigated the relation between sports participation, body size, and postural control in children between 3 and 11 years of age. To explore this question, children's body sway was measured across multisensory conditions manipulating visual input (the presence versus absence of visual information) and proprioceptive input (varying stance widths), with postural sway in these conditions then related to reports of children's sports participation, and anthropometric measures. Corroborating well-known findings, postural sway was systematically influenced by multisensory factors, with the removal of visual information and narrower stance widths decreasing postural stability. Of more novelty, postural sway in the most stable stance, but without vision, was significantly predicted by measures of sports participation and body size variables, with these factors contributing independently to this prediction. Moreover, the impact on postural sway of having visual input, relative to removing visual input in unstable stances, was significantly predicted by sports participation in activities stressing changing stances and bases of support (e.g., dance, martial arts). Generally, these findings support multisensory and dynamic systems theories of perceptual-motor behavior, and also support sports specificity effects in assessments of the relation between posture and sports.

## 1. Introduction

Maintaining one's posture is critical for successful interactions in the world. As such, understanding how posture is controlled is of fundamental importance, and includes an assortment of reflexive [1], muscular [2], biomechanical [3], and multisensory factors [4],[5]. Such factors have been found to similarly influence postural control in children, with research again focusing on muscular, biomechanical, and multisensory influences [6]–[11]. One aspect that has been curiously understudied, however, involves influences such as social factors, and individual difference factors ranging from basic body size parameters to experiential components, such as training.

Theoretically, one framework that could encompass such factors alongside biomechanical and multisensory influences is found in dynamical systems theory [12],[13]. Simply described, dynamical systems theory describes “...behavior as the emergent product of a self-organizing multicomponent system evolving over time” (Perone & Simmering, 2017, p. 44), and emphasizes that among these components, no single factor is pre-eminent in its influence, with any of the components functioning as a critical, rate-limiting factor at different developmental times [14]. Systems approaches have provided powerful explanatory frameworks for motor behavior [15], cognitive abilities [16], and social skills [17]. Accordingly, this framework is ideal for incorporating non-sensory factors, such as motivation [18], social influences [19], and individual difference factors such as body size and experiential influences into an understanding of postural control.

This is not to say that the impact of explicit training on posture has not been investigated. Indeed, work in sport and exercise has a rich history of exploring the relation between posture and performance in sports, tending to focus on whether varying “levels” of sport performance differentially influence postural control ([20],[21], see reviews by [22],[23]). For instance, some work has examined whether there are differences in postural control between trained athletes and non-athletes [24]–[27]. For instance, Golomer, Crémieux, Dupui et al [26] found that dancers were generally more stable than non-dancers, and were less dependent on visual information for posture. Relatedly, Paillard and Noé [28] observed differences in posture between professional and amateur soccer players. Although this literature has produced mixed results, overall the preponderance of studies suggests that athletic training increases stability in different contexts [23].

A second question explores whether there are differences in postural control arising from the specific sports in which participants partake [20], given that varying athletic activities make different demands on posture. In keeping with this hypothesis, this work has demonstrated that expertise in different sports is associated with different forms of postural control [25],[27]. Perrin, Deviterne, Hugel et al. [27], for instance, found that judoists and dancers showed more stable posture than nontrained participants with visual input, and that judoists outperformed dancers without visual input. Overall, these findings have led to the idea that “... athletes of different specializations have a better ability to maintain balance in specific conditions” (Paillard, 2014, p. 1019).

Finally, there have been multiple investigations looking at the relation between sports participation and posture in a developmental context. For instance, Tsang, Wong, Fu et al. [29] observed that regular Tai Chai practice in elderly participants was associated with improved postural control relative to non-Tai Chi participants. Other work has focused on children [24],[30]. For instance, Salhi, Ghroubi, Rebai et al. [30] found that 5- to 6-year-olds who had received circus training showed increased postural control, relative to children without such experience. One limitation to such work is that it says little about general developmental processes, although Kiomourtzoglou, Derri, Mertzanidou et al. [31] did compare variously aged elite rhythmic gymnasts to age and body-sized matched controls. Ironically, these authors found that general body size variables, which change systematically with age, were not the driving factor in differences between these participants.

Research in sports and posture also contains inherent limitations. One such constraint is a limitation on the multisensory factors studied, with the focus being primarily on visual input. One notable exception involves comparisons of unipedal versus bipedal stance [23],[25], with stance differences viewed as varying proprioceptive (somatosensory) input [32]. Although stance is not commonly thought of as providing proprioceptive input, because changes in stance width modify percepts of one's body and joint positions [33],[34], such modifications can be seen as varying proprioceptive information [32]. A second constraint in such work is the lack of consideration of general developmental mechanisms as potentially underlying changes in the relation between posture and sports. One such developmental influence involves basic anthropometric factors such as body size. Previous work with children has found that postural control is predictable from anthropometric parameters in terms of body mass [35], and participant height and leg length [9]. Given such findings, investigating developmental parameters within the context of sports participation represents an important extension to such questions.

As a final point, the general focus on sport-specific effects on posture misses the opportunity to explore whether all athletic activity leads to general differences in motor control, or whether such effects are truly sport specific. One way of addressing this distinction involves exploring athletic engagement in populations who routinely engage in multiple sports without performing at an elite level, to see if diversity of athletic activity in and of itself can influence motor control. Investigating a multi-sport approach is most easily accomplished within a developmental context, given that it is common for children to participate in multiple athletic activities on a continuous basis. The goal of the current project was to address these limitations regarding multisensory postural control and the relation between sports and posture.

## 2. Materials and methods

### 2.1. Participants

**Figure 1.**
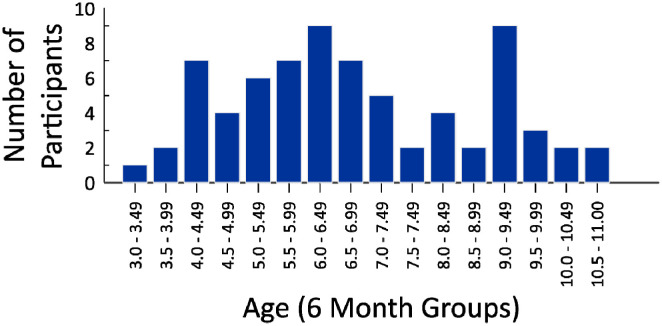
Distribution of participants across age (in years), grouped into 6-month intervals.

Explicit exclusion criterion involved the occurrence of head injury in children, or a reported developmental disability involving motor control. The final sample of participants included 72 children (32 females) from 3 to 11 years (*M* age = 6.77 years, *SD* = 1.96). An additional 11 participants were recruited, but their data were not analyzed due to experimenter error (2), refusing to comply with experimental instructions (3), being a child with special needs (1), and providing sway measures greater than 2.5 SDs from the mean of the full sample (5). Children were recruited from the local community, and were compensated for participating with a toy. Figure 1 presents the age distribution (in years) of participants, broken down into 6-month intervals. This research was approved by the University of Toronto Ethics Review Board (protocol #22601), and was in accordance with the 1964 Declaration of Helsinki. All parents provided written informed consent for their children to participate in this study.

### 2.2. Materials

Centre of pressure (CoP) measurements corresponding to body sway were captured using a force plate (Kistler Model 9260AA). The force plate was connected to a Windows based computer, and data were sampled using the Kistler Bioware® software, at a sampling frequency of 50 Hz. This sampling frequency has been employed in other studies in the lab, and can reliably detect changes in postural sway as a function of multisensory manipulations [10],[32],[36].

### 2.3. Experimental conditions

The study contained two experimental conditions manipulating the presence versus absence of different sensory inputs. The first variable involved the availability of *visual information*, and consisted of “eyes open” trials producing visual input, and “eyes closed” trials removing visual input. The second variable manipulated the available *proprioceptive information*, produced by having participants adopt three different stances. In the “shoulder width” stance, participants adopted a natural base of support with feet positioned roughly one shoulder's width apart. In the “feet together” stance, participants adopted a narrowed base of support in the mediolateral axis of sway. And in the “tandem” stance, the feet were aligned in the anterior-posterior axis of the body, with the heel of one foot touching the toe of the other foot. For the majority of children, the right foot was in front of the left foot, with the exception of a handful of children who specifically requested a reversed foot placement. Cheung and Schmuckler [32] demonstrated that these conditions systematically decrease postural stability in adults.

### 2.4. Design

The study employed a within-subjects factorial design, crossing the *visual information* condition and *proprioceptive information* condition, producing six trials per participant. Trials were blocked by the *proprioceptive information* condition, with these blocks presented in different random orders for participants. As an aside, these manipulations represent a subset of experimental variations employed in a larger research project investigating the impact of a wider array of multisensory input on children's postural control (see [37] for a discussion). The data discussed in this report were extracted from this dataset as (1) they were most germane to the research questions under investigation, and (2) the findings from the larger data set have yet to be published.

### 2.5. Procedure

Upon entering the lab, the experimental conditions were described, anthropometric measurements were taken, and parents were interviewed as to their children's sports participation (see below). Children then removed their shoes, and were instructed as to how to stand on the force platform.

During experimental trials children stood with arms and hands at their sides, with instructions to close or open their eyes (order randomized) provided at the beginning of each trial. All trials were 20 seconds in length, with approximately 5–10 seconds between trials. After each block, participants received instructions regarding the feet position (order of stance randomized) for the next block of trials. After completing all blocks, parents were debriefed as to the purposes of the experiment. The experimental session lasted about 25–30 minutes.

### 2.6. Center of Pressure (CoP) preprocessing

Experimental records of sway in anterior-posterior and mediolateral dimensions were used to calculate resultant distance (RD) values. Because RD values most comprehensively characterize postural sway, these values were employed in all analyses. RD values were preprocessed using a 4^th^ order Butterworth low-pass filter with a 5 Hz cut-off,^1^ and were used to calculate multiple postural stability measures, including time-domain distance (e.g., mean distance and velocity), time-domain area (e.g., 95% confidence circle), and time-domain hybrid measures (e.g., sway area). Ultimately, mean velocities were employed in analyses given that this measure is representative of the time-domain parameters, has been employed in previous work [10],[32], and is sensitive to differences arising from experimental manipulations and age [38].

### 2.7. Quantification of postural control, anthropometric, and sports participation measures

Postural stability was explored by analyzing the mean velocity of sway (cm/s), which most directly assess the impact on posture of visual and proprioceptive manipulations. Anthropometric data included measures of standing and sitting height, leg length, waist and shoulder widths, and weight. Heights (in inches) were measured using a tape measure attached to a nearby wall, with leg length calculated by subtracting sitting height from standing height. Waist and shoulder widths (in inches) were calculated by wrapping a tape measure around participants' waist and shoulders. Weight (in lbs) was measured on a standard bathroom scale.

Sports participation was quantified by asking parents about the sport activities in which their children engaged on a regular basis. Specifically, parents were asked about the frequency with which children participated in a given sport on a weekly basis (hours/week), and the duration of time (years) in which they had participated in that sport. Sports participation was ultimately categorized into four groups—ball sports, dance, swimming, and other. These categories were partly practical (the first three were the most common reported by parents) and partly based on divergent postural demands. Dance and swimming represent sports in unique postures, with dance combining bipedal and unipedal stances that are dynamic and static, and swimming an activity with literally no upright postures. Ball sports represent an aggregation of common sports, including soccer, baseball (and t-ball), basketball, hockey, and badminton, all of which emphasize bipedal, dynamic postures. The “other” category consisted of the remaining athletic activities in which children engaged, including primarily martial arts (karate, judo, taekwondo, mixed martial arts), gymnastics, horseback riding, cross-country running, track and field sports, skiing, yoga, and bicycling. With the exception of martial arts, many of the sports in this final category were specific to only one or a few individual participants.

Sports participation scores for all categories were calculated using the following formula: *Category (i.e., ball, dance, swim, other) sports participation (hrs)* = Sports frequency (hrs/wk) × Sports duration (yrs) × 52 (wks/yr). All participants received a score for each category, with children getting a score of 0 for sports categories in which they did not participate. Individual category sports participation scores were used in subsequent analyses, and were also combined into a *total sports participation* score (in hours) by adding together these four categories. Finally, the *total number of sports* was calculated by summing the number of sports in which children participated; this measure represents sports variability, as opposed to sports experience.

## 3. Results

**Figure 2.**
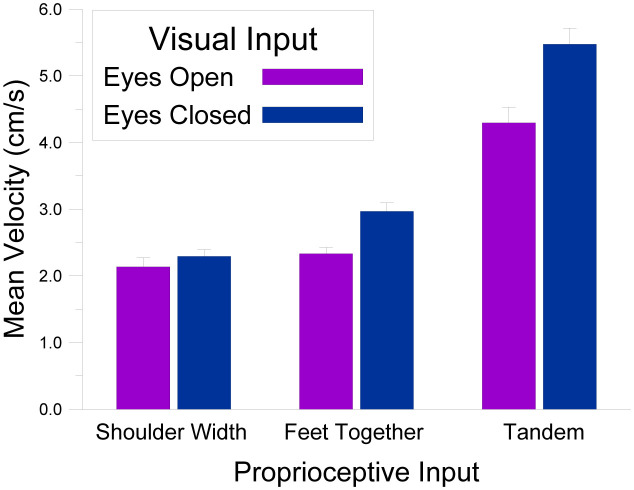
Mean velocity values as a function of the *Visual Input* (eyes open, eyes closed) and *Proprioceptive Input* (shoulder width stance, feet together stance, tandem stance) manipulations.

Although the principal goal of this work was to relate postural stability to sports participation and anthropometric measures, it is important to examine these measures in and of themselves to ensure that any differences in sway as a function of the experimental manipulations are consistent with the general literature on multisensory influences in postural control in adults. Accordingly, mean velocities were analyzed in a two-way ANOVA (all statistical analyses were conducted in Jamovi 2.2.5), with within-subjects factors of *Visual Information* (eyes open, eyes closed) and *Proprioceptive Information* (shoulder width stance, feet together stance, tandem stance). This analysis produced main effects for *Visual Information*, *F*(1, 70) = 45.91, *MSE* = 1.05, *p* < .001, *np^2^* = .384, and *Proprioceptive Information*, *F*(2, 140) = 128.54, *MSE* = 2.27, *p* < .001, *np^2^* = .647, and a significant interaction between the two, *F*(2, 140) = 11.52, *MSE* = 0.08, *p* < .001, *np^2^* = .141. Figure 2 shows this interaction and reveals that the presence of visual information (*M* = 2.92, *SE* = .12) increased postural stability (lower velocity values) relative to no visual information (*M* = 3.58, *SE* = .11). Similarly, shoulder width stances (*M* = 2.22, *SE* = .10) led to the greatest postural stability, followed by feet together stances (*M* = 2.65, *SE* = .09), and then tandem stances (*M* = 4.88, *SE* = .21). Finally, the impact of having visual input increased in unstable stances, with a significant difference between visual conditions for feet together, *t*(70) = 5.18, *p* < .001, and tandem stances, *t*(71) = 5.16, *p* < .001, but no difference for shoulder width stances, *t*(71) = 0.90, *n.s*. Overall, these findings converge with the multisensory postural control literature with adults [32].

**Figure 3.**
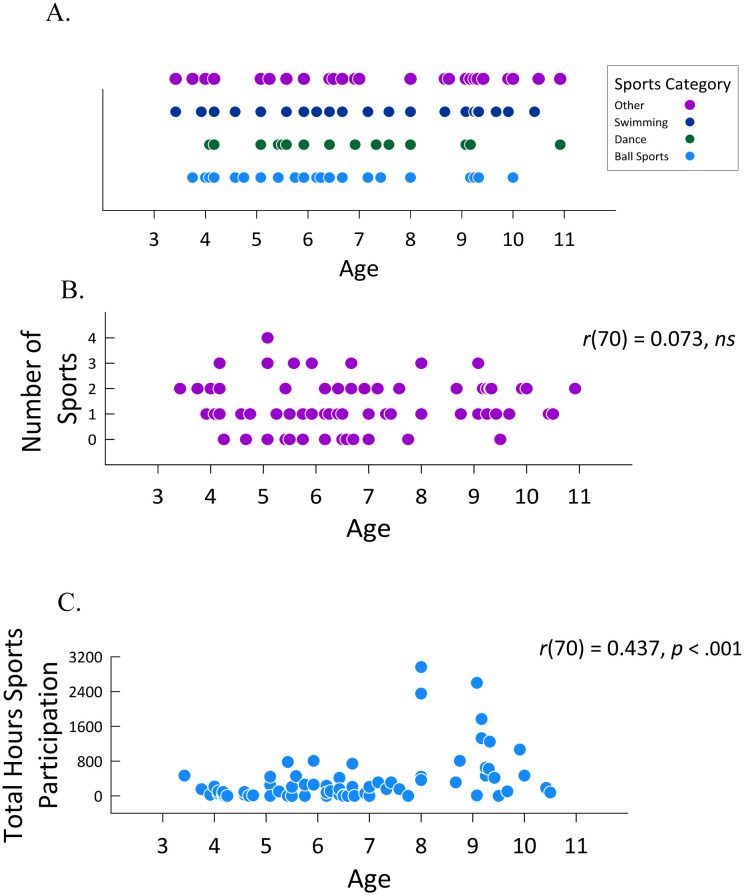
Age distribution within each of the sports categories (A), as a function of total number of sports played (B), and the total hours of sports participation (C).

Additional preliminary analyses examined the general prevalence of sports participation as a function of the individual categories, and across age within the individual categories. Specifically, 26 children participated in ball sports, 16 in dance, 26 in swimming, and 32 in the other category; note that these values do not sum to 72 (the number of participants in this study) as children could participate in more than one category. Additionally, 13 children did not participate in any category of sports, 28 participated in one category, 22 participated in two categories, 8 in three categories, and 1 in all four categories; note that these values do sum to 72. Figure 3A presents a breakdown of the age distribution of children in each of the sports categories. As can be seen, within each category, age was generally evenly distributed. Subsequent analyses examined the relation between age and sports participation, correlating age with both the total number of sports in which children participated (Figure 3B) and the total sports participation score (Figure 3C). Interestingly, age did not correlate with the total number of sports played, *r*(70) = .07, *ns*, but it did correlate with the total sports participation score, *r*(70) = .44, *p* < .001. Thus, although increasing age did not influence the breadth of sports participation, it (not surprisingly) affected the extent of sports participation.

Turning to the principal analyses for this study, the first step in relating postural stability with sports activity and anthropometric measures involved calculating intercorrelation matrices within each of these sets of measures. Understanding the inter-relations within each set of factors is critical for properly assessing the relations between these factors. These correlation matrices appear in Table 1, and reveal that each of these sets of measures were logically inter-related.^2^ Thus, postural control measures across shoulder width and feet together stances were strongly intercorrelated, with more sporadic correlations for tandem stance. Similarly, anthropometric measures, with the exception of weight, were strongly intercorrelated. Sports participant measures, in contrast, were unsurprisingly unrelated, although the individual measures were related to the overall sports participation values.

**Table 1.** Intercorrelation matrices for the postural control measures (mean velocity) across different stance width conditions, sports participation measures, and anthropometric measures.

*Postural Control Measures (condition)*		Shoulder Width	Feet Together		Tandem	
		Eyes Closed	Eyes Open	Eyes Closed	Eyes Open	Eyes Closed
Shoulder Width	Eyes Open	.511****	.637****	.292*	.177	.060
	Eyes Closed		.418****	.419****	.158	.016

Feet Together	Eyes Open			.407****	.296*	.212^B^
	Eyes Closed				.231^A^	.336***

Tandem	Eyes Open					.566****
	Eyes Closed					

*Sports Participation Measures*		Dance	Swimming	Other Sports	Total Sports Participation	Number of Sports

	Ball Sports	.211^C^	.061	-.033	.508****	.253*
	Dance		-.002	.197	.454****	.191
	Swimming			.087	.286*	.360***
	Other Sports				.802****	.418****
	Total Sports Participation					.547****

*Anthropometric Measures*		Leg Length	Weight		Waist Width	Shoulder Width

	Height	.932****	-.060		.722****	.829****
	Leg Length		-.041		.658****	.792****
	Weight				-.011	-.048
	Waist Width					.741****

* *p* < .05; ** *p* < .01; *** *p* < .005; **** *p* < .001; ^A^ *p* ≤ .06; ^B^ *p* ≤ .07; ^C^ *p* ≤ .08; ^D^ *p* ≤ .09

Of central importance, Table 2 presents the correlations between postural control measures and the sports activity and anthropometric measures. Significance for the correlations in this table are indicated in two ways. First, significance levels for the uncorrected correlations are indicated via superscripts attached to the correlation values, with these superscripts defined in the legend at the bottom of the table. Second, correlation significances were corrected for multiple comparisons using Benjamini and Hochberg's [39] false discovery rate procedure. Significant correlations after these corrections are presented in bold in this table.

**Table 2.** Correlations between posture, sports participation and anthropometric measures.

Mean Velocity Values						
	Shoulder Width		Feet Together		Tandem	
	Eyes Open	Eyes Closed	Eyes Open	Eyes Closed	Eyes Open	Eyes Closed
*Sports Participation*						
Ball Sports	-.038	-.103	-.115	.126	.041	-.041
Dance	-.063	-.272*	-.196	-.272*	-.126	-.009
Swimming	-.122	.043	.106	.040	-.076	-.112
Other Sports	-.133	-.284*	-.050	-.191	-.091	.138
Total Participation	-.155	-.312**	-.113	-.133	-.090	.062
Number of Sports	-.089	-.265*	-.107	-.141	.017	.154

*Anthropometric*						
Height	-.310**	-.310**	-.101	-.075	-.211^C^	.074
Leg Length	-.223^A^	-.261*	-.006	-.094	-.175	.085
Weight	.304**	.011	.149	-.060	.128	-.077
Waist Width	-.220^B^	-.203^D^	-.082	-.162	-.128	-.008
Shoulder Width	-.162	-.148	.035	-.032	-.191	.015

* *p* < .05; ** *p* < .01; *** *p* < .005; **** *p* < .001; ^A^ *p* ≤ .06; ^B^ *p* ≤ .07; ^C^ *p* ≤ .08; ^D^ *p* ≤ .09

Overall, (some) postural control measures were correlated with both sports participation and anthropometric variables. Specifically, mean velocities in the shoulder width, eyes closed condition correlated with the total hours of participation, *r*(70) = −.312, *p* < .01, and the number of sports, *r*(70) = −.265, *p* < .05, and the mean velocities in the shoulder width and feet together, eyes closed conditions correlated with dance experience, both *r*'s(70) = −.272, *p* < .05. Thus, as sports experience increased postural stability increased.

Table 2 also shows that the anthropometric variables correlated with postural stability. Specifically, there were significant correlations between mean velocities in both shoulder width conditions and standing height, leg length, and body weight, with marginally significant correlations for waist circumference.

Subsequent analyses explored the joint contributions of sports participation and body size measures to predictions of postural sway. Specifically, postural stability was predicted from the total hours of participation and participant height; these factors were chosen as representative of the sports participation and anthropometric categories discussed earlier. Out of these analyses there were significant predictions for the two shoulder width stance conditions, eyes open *R* = .310, *p* = .031, and eyes closed *R* = .363, *p* = .008. For the eyes open condition this effect was driven by participants' height, ß = −.305, *p* = .022, and not sports participation, ß = −.011, *p* = .931, whereas in the eyes closed condition, both factors contributed marginally, with participation hours, ß = −.213, *p* = .098, and height, ß = −.209, *p* = .104.

Finally, postural stability was predicted from the specific sports experience categories, in conjunction with anthropometric factors. Because sports participation was a factor only in the shoulder width, eyes closed condition, this analysis focused solely on this measure. Accordingly, mean velocities in this condition were predicted from the participation categories of ball sports, dance, swimming, and other sport activities, along with participants' height. This analysis produced a multiple *R* = .431, *p* = .016, with dance experience, ß = −.216, *p* = .068, and height, ß = −.253, *p* = .052, primarily contributing to this prediction.^3^ Overall, this analysis reveals that it is possible to parse the relation between posture and sports participation into specific sports activities, in keeping with the idea of specificity effects of athletic experience on balance control.

## 4. Discussion and conclusion

The principal novel finding in this study was that postural control was related to both sports participation and anthropometric measures. Specifically, posture in the most stable stance without vision was related to increasing sports participation and increasing body size variables, the impact of removing vision in the most unstable stance was related to increasing sports participation and body size values. Moreover, these two sets of factors contributed independently to stability. To our knowledge, this is one of the first demonstrations of an impact of sports experience on posture outside of an elite athletic perspective context, and one of the first examinations of this relation within a framework of general development factors.

Theoretically, the role for sports experience in postural control suggests that the oft-discussed “multisensory” framework for postural control [4],[5] represents an incomplete characterization of the influences impacting on stability. Rather, a dynamic systems approach [12],[13],[14] provides a more comprehensive framework in which multisensory factors can co-exist with sports participation and body size factors. One advantage to this framework is that it emphasizes that the end state behavior is ultimately the systems product of all of factors, with the relative importance of the different components varying as a function of environmental conditions, developmental stage, and so on. More generally, this framework allows for a host of various influences in characterizing posture and its development.

Another theoretical implication of these findings involves support for the sport specificity hypothesis [20]. Specifically, it is notable that sway was related to overall sports participation, and that this influence could be disentangled to highlight the role of dance experience and the “other” category that consisted largely of martial arts experiences. As previously noted, these two categories are distinctive in their emphasis on maintaining posture across stances varying in width and the interchanging use of unipedal and bipedal stances. One (speculative) explanation is that athletes with heightened experience across varying stances have learned to control their balance across such changes by employing differential use of multisensory information. Indeed, this explanation fits well with work on sensory reweighting in postural control [40],[41], in which the reliance on an information source changes as a function of its reliability.

Another point of importance arises from the development framework of this project. In this regard, it is notable that the role of body size in posture has been curiously understudied in the literature, with the majority of such work focusing on the impact of obesity on balance. Accordingly, exploring a wider range of body size variables within a developmental context represents a significant innovation in this work. Even more important is the fact that the relation between body size and posture was actually opposite to what has been observed with adults. Specifically, this work found that increasing body size was related to increased postural stability, a result opposite to what has been seen in adults [32]. One explanation for this difference is that for children this relation represents increasing motor coordination across development, an idea consistent with the fact that children do not achieve adult levels of postural control until mid-childhood, roughly 6–8 years [42],[43]. Accordingly, body size in this context is really a proxy for overall maturation in children. This relation thus contrasts with adults in which balance is understood with reference to an inverted pendulum model [3], with taller adults seen as more inherently unstable pendulums.

Finally, and with respect to the developmental implications of this work, one curious aspect of the current project is that, throughout the developmental analyses, there has generally been no form of control for “age” per se, separated from the various experiential and anthropometric factors. This lack of controlling for age could be seen by some as potentially problematic, ignoring a critical factor that could be driving some of the observed relations. In assessing such a concern, it is important to remember that age in and of itself is not, in any sense, a causal variable in any direct fashion (see [44] for a discussion of this point). Rather, age is generally used as placeholder for representing unarticulated and unrecognized cognitively-based, bodily-based, or experientially-based factors that vary in accordance with changing age. In the current case we have gone to great pains to identify and assess a host of potential underlying causal factors driving postural stability. Accordingly, assessment of, and controlling for, “age” in and of itself in this context is neither necessary nor informative. This does not mean, of course, that there may not be factors other than those we have identified that might be contributing to postural stability in this work. Indeed, the point of this study in the first place was to highlight and assess previously overlooked factors (sports experience) that might be related to postural stability; it would be significant hubris to assume that other unidentified factors are not also influencing posture in this work. However, the impact of such factors would benefit from explicit identification and assessment. Relegation of such influences to a non-specific, non-causal “age” factor simply does not provide meaningful insight.

Most fundamentally, this project has highlighted the importance of expanding current framework for characterizing posture by integrating non-sensory components. On its most general basis, these results have intriguing implications for both how we might profitably continue to examine postural control, and how we conceptualize the perceptual-motor processes underlying this critical behavior.

## Footnotes

^1^ Frequently researchers trim an initial first few seconds from trials to avoid “settle-in” effects for participants. In fact, preliminary analyses employing such a trimming procedure failed to produce any significant differences in sway measures relative to the complete, untrimmed data set. Accordingly, results employing the fully intact data set are reported here.

^2^ Given the large number of correlations presented in Tables 1 and 2, there is a concern about the possibility of false positive correlations. Accordingly, the correlations for Table 2 were corrected for multiple comparisons (see text). However, the correlations presented in Table 1 were not corrected, given that the primary goal of these analyses was to simply demonstrate expected relations between these measures, and not to assess the strength of these relations *per se*.

^3^ A subsidiary analysis predicting velocity values from only the sports participation variables also revealed a significant fit, *R* = .372, *p* = .039. This analysis also found that, along with dance contributing to this prediction, ß = −.207, *p* = .085, the other sports participation category was an important factor, ß = −.251, *p* = .035.
